# Delineating the Psychic Structure of Substance Use and Addictions, from Neurobiology to Clinical Implications: Ten Years Later

**DOI:** 10.3390/jcm9061913

**Published:** 2020-06-18

**Authors:** Pier Paolo Pani, Angelo G. I. Maremmani, Matteo Pacini, Emanuela Trogu, Gian Luigi Gessa, Pedro Ruiz, Icro Maremmani

**Affiliations:** 1 Social-Health Services, Azienda Tutela Salute Sardegna (Sardinia Health Trust), 09128 Cagliari, Italy; pierpaolo.pani@atssardegna.it; 2 Department of Psychiatry, North-Western Tuscany Local Health Unit, Tuscany NHS, Versilia Zone, 55049 Viareggio, Italy; angelo.maremmani@uslnordovest.toscana; 3 Association for the Application of Neuroscientific Knowledge to Social Aims (AU-CNS), 55045 Pietrasanta, Italy; 4 PISA-School of Experimental and Clinical Psychiatry, 56100 Pisa, Italy; 5 G. De Lisio Institute of Behavioral Sciences, 56100 Pisa, Italy; paciland@virgilio.it; 6 Department of Psychiatry, ASSL, 09121 Cagliari, Italy; troguemanuela@gmail.com; 7 Emeritus of Neuropharmacology, University of Cagliari, 09124 Cagliari, Italy; lgessa@unica.it; 8 Department of Psychiatry and Behavioral Sciences, Baylor College of Medicine, Houston, TX 77030, USA; pruizmd@outlook.com; 9 Vincent P. Dole Dual Disorder Unit, 2nd Psychiatric Unit, Santa Chiara University Hospital, University of Pisa, 56100 Pisa, Italy

**Keywords:** psychopathology specific to substance use disorders, addiction, psychiatric comorbidity

## Abstract

The diagnosis of substance use disorder is currently based on the presence of specifically identified behavioral symptoms. In addition, other psychiatric signs and symptoms accompany addictive behavior, contributing to the full picture of patients’ psychopathologic profile. Historically, such symptoms were confined within the framework of “comorbidity”, as comorbid psychiatric disorders or personality traits. However, an alternative unitary view of the psychopathology of addiction, inclusive of related psychiatric symptoms, has been claimed, with the support of epidemiological, neurobiological, and neuropsychological evidence. In the present article, we highlight the research advancements that strengthen this unified perspective. We then give an account of our group’s definition of a specific SCL-90-based construct of the psychopathology of addiction. Lastly, we discuss the benefits that can be expected to be acquired in the evaluation and treatment of patients with a longitudinal approach including psychological/psychiatric predisposing features, addictive behavior, and psychiatric manifestations.

## 1. Introduction

The diagnosis of substance addiction is nowadays based on the presence of specifically identified behavioral symptoms. In addition, other signs and symptoms (such as novelty seeking, irritability, restlessness, impulsivity, diminished interest in activities, dysphoria, boredom, depression, and attention and concentration difficulties) usually accompany addictive behavior, thus contributing to the full picture of their psychopathological profile, and, of course, to persistence in the use of substances. These symptoms, which belong to the domains of anxiety, mood, and impulse control, do not find space within the diagnostic criteria of substance use disorder, being historically confined within the framework of “comorbidity”, as comorbid psychiatric disorders or personality traits.

About ten years ago, we challenged this divisive approach, proposing a unitary view of the psychopathology of addiction, by making it inclusive of related psychiatric symptoms [1]. In this article, we assert that psychiatric symptoms displayed by patients dependent on substance abuse are closely associated with addiction, as demonstrated by the high frequency of the coexistence and by the evidently shared neurobiology and neurophysiology. We support this unitary view by referencing abundant epidemiological, neurobiological, and neuropsychological evidence.

Given this background, and the persisting uncertainty on the appropriate collocation of psychiatric symptoms—in deciding whether they belong to the addictive disorder or to comorbidity—we approach the study of psychopathology of addiction from a low inference level, looking to the symptoms manifested by patients, instead of to the pre-defined syndromes of the current nosography.

In the present article, we give an account of the contribution made by our research group to the definition of an SCL-90-based specific psychopathology of addiction. We then discuss the benefits that can be expected to be gained in the assessment and treatment of patients by adopting a longitudinal approach to patients, inclusive of psychological/psychiatric predisposing factors, addictive behavior, and psychiatric manifestations.

## 2. Nosography: Critical Issues

Despite the scientific advances achieved in neurobiology, genetic, epigenetic, and neuropsychology, which testify to strong communalities and dynamic interactions between addictive disorders on the one hand and other psychiatric conditions on the other hand, nosographic systems, such as the DSM system, confine the diagnosis of addictive disorders within a narrow range of behavioral manifestations. Even the DSM-5, although adding craving to the diagnostic criteria, does not include in its description of the disorder other non-behavioral symptoms, thus reserving the diagnosis of associated psychiatric conditions to other separate domains such as that of mood, anxiety, and impulse dysregulation.

However, given the advances made in scientific research, the current categorical approach to nosography may be destined to be overcome by a more appropriate etiopathogenetic classification system of mental disorders consistent with the existence of specific neurobiological processes that support a variety of psychiatric conditions/disorders that share the same pathophysiology. These pathophysiological traits may be reinterpreted by the clinic as transdiagnostic psychological/psychiatric defects and vulnerabilities that play the role of “endophenotypes”. In particular, the RDoC program developed by the US National Institute of Mental Health (NIMH) has been made available to researchers in order to pursue new ways to classify mental disorders by basing them on the transdiagnostic domains, constructs, and subconstructs that link neurobiological systems, together with their subunits (genes, molecules, neurons, and neural circuits), to different but related functional and dysfunctional conditions [2,3]. Interestingly, the RDoC provides a dimensional perspective on psychiatry, ranging from normal to pathological, thus including diagnostically subthreshold psychological/psychiatric conditions, psychological/psychiatric precursors, and predisposing factors. Prime examples of these neurobiologically based systems considered in the transdiagnostic RDoC approach to addiction pathogenesis, and in mood and impulse control dysfunctions, are reward-processing systems and inhibitory systems [4,5,6,7,8,9].

### 2.1. Applying a Low-Inference Observation Level

In the interim, while the search for an etiopathogenetically and pathophysiologically grounded nosography is progressing, efforts to model a unitary psychopathology of addiction have been accomplished by looking at the aggregation of psychological/psychiatric symptoms exhibited by subjects with substance use disorders [10,11,12,13,14,15]. Following this approach, our research group has tried to identify, in patients with an addictive disorder, homogeneous psychological/psychiatric dimensions resulting from the aggregation of answers given to the Self-Report Symptom Inventory (SCL-90) questionnaire [13,14].

The SCL-90 is a self-report questionnaire used to measure psychological/psychiatric symptoms. It comprises 90 items and allows the evaluation of psychological/psychopathological severity across nine dimensions including both internalizing and externalizing symptoms.

All studies included in our line of research were conducted according to the WMA Declaration of Helsinki—Ethical Principles for Medical Research Involving Human Subjects. Subjects examination was preceded by informed consensus and local ethics committees were involved in approval.

**Figure 1:**
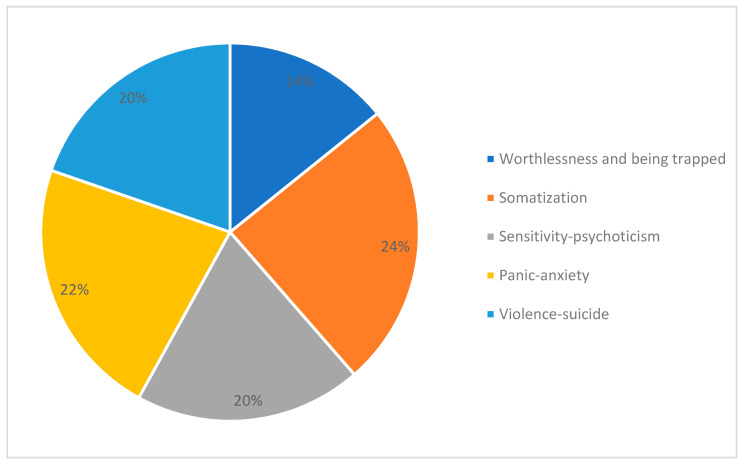
Patients assigned to psychopathologically dominant groups according to Z-scores for each of the five clusters obtained by the exploratory principal component factor analysis (PCA) applied to 1055 SCL-90 questionnaires filled in by patients with opioid dependence at the time they started to take opioid agonist medications [14].

As first step in this line of research, we performed an exploratory principal component factor analysis (PCA) on 1055 SCL-90 questionnaires filled in by patients with opioid addiction at the start of their treatment with opioid agonists (OAT). As result of this analysis, instead of the known nine factors identified in psychiatric patients, we obtained a five-factor solution. The first factor identified a depressive “Worthlessness/Being Trapped” dimension with: feelings of worthlessness; feeling lonely, hopeless about the future; and a dominant sensation of being trapped or caught. The second factor reflected a “Somatic Symptoms” dimension, with: soreness of muscles; heaviness in arms; hot or cold spells; weakness; nausea or upset stomach; and insomnia. The third selected a “Sensitivity/Psychoticism” dimension, with: sensation of being watched or talked about by others; other people knowing your private thoughts; sensations to be perceived as unfriendly or disliked; and having thoughts that are not your own. The fourth was a “Panic Anxiety” dimension, with: feeling fearful; feeling afraid in open spaces afraid to leave home alone; spells of terror or panic; and feeling afraid to travel. The fifth was a “Violence/Suicide” dimension, with: shouting/throwing things; urges to break or smash things up; outbursts that you cannot control; and thoughts of death or dying. According to the highest Z-score obtained for each factor, we distributed participating subjects into five samples (called “dominant groups”). The most numerous groups of patients were those distinguished by somatic symptoms. The second most numerous dominant group was the panic anxiety group, followed by the violence/suicide group, the sensitivity/psychoticism group, and, lastly, the worthlessness/being trapped group (see Figure 1).

**Figure 2:**
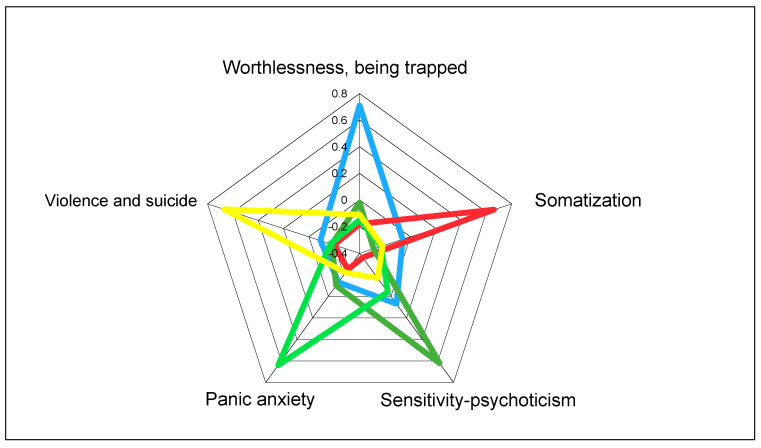
Independence of psychopathological dimensions identified by exploratory principal component factor analysis (PCA) applied to 1055 SCL-90 questionnaires answered by patients with opioid dependence at the onset of the treatment with opioid agonists: highest Z-scores obtained by patients on each dimension [14].

Each of these five dimensions proved to be independent of the others. As shown in Figure 2, when looking at the highest Z-scores obtained by patients among the five dimensions, each dominant group obviously obtained the highest score in its own dimension, while the scores recorded in all the other dimensions were very low (below zero). Only patients grouped in the “Worthlessness/Being Trapped” dimension obtained scores slightly above zero in the “Sensitivity/Psychoticism” dimension. These groups appear to be distinct, and they fail to display any significant overlap.

We replicated this first investigation with another sample of 1195 patients with opioid dependence at the start of their residential treatment in a Therapeutic Community (TC). The confirmatory factor analysis (PCA) carried out on the SCL-90 filled in by these patients generated a five-factor solution, confirming the dimensions obtained in patients at the beginning of OAT: “Worthlessness/Being Trapped”, “Somatic Symptoms”, “Sensitivity/Psychoticism”, “Panic Anxiety”, and “Violence/Suicide” [16]. Moreover, on applying a logistic regression analysis to the merged sample (including both OAT and TC patients), we observed an association of “Somatic Symptoms” and “Violence/Suicide” dimension severity with OAT (Somatization OR 0.47 (CL 95% 0.32–0.69); Violence/Suicide OR 0.66 (CL95% 0.43–0.99).

Following this line of enquiry, several further studies were carried out to ascertain whether this psychopathological structure can be considered a stable trait, or a variable state conditioned by potential confounding factors. Among these factors, we considered demographic and clinical characteristics. Regarding demographic ones, patients belonging to the five groups did not turn out to differ on gender, marital status, education, income, living situation, or length of addiction. The only sociodemographic difference that was observed was in age; older patients with opioid dependence were, in fact, characterized by their high scores for the Somatic Symptoms and Worthlessness/Being Trapped symptom sets [13,14,17]. We went on to verify the impact of ethnicity, by comparing 66 Slovenian with 66 Italian matched heroin-dependent patients, and 30 migrants with 30 matched Italian heroin-dependent patients, without observing any statistically significant differences in the percentage of the five SCL-90 defined dimensions [18,19].

With respect to potential clinical confounding factors, we took into account: presence of active heroin use, presence of psychiatric problems, response to stress, and consumption of alcohol or cocaine. The first of these studies, involving 1015 participants, was planned to find out if opioid addicts who had undergone detoxification (DTX) and patients not detoxified (NDTX) from heroin differed in their SCL-90 psychopathological profile [20]. The severity recorded for all the SCL-90 factors was higher in NDTX versus DTX patients. All the differences found were statistically significant. However, the severity of “Somatic Symptoms” was the only factor identified by the stepwise discriminant analysis as capable of differentiating NDTX from DTX patients. Despite the statistical significance, the percentage of originally grouped cases that proved to have been correctly classified was moderate (64%) (see Table 1).

Regarding psychiatric comorbidity, in a sample of 455 heroin-dependent patients, we did not observe any statistically significant differences in the presence of dual diagnosis between patients allocated to any of the five dimensions. However, heroin addicts with bipolar spectrum-related conditions were significantly better represented in the Violence/Suicide group than in the Sensitivity/Psychoticism one [17]. We also looked at the differences in the psychopathological profiles defined by SCL-90 between patients dependent on opioid with (PC-HA) or without (NPC-HA) ascertained lifetime psychiatric problems [21]. According to this investigation, all the SCL-90 severity scores (total sample numbering 1195) were significantly higher in PC-HA than in NPC-HA subjects. In addition, when applying stepwise discriminant analysis, the only factor discriminating NPC-HA from PCHA patients was the severity of “Panic Anxiety” and, in a similar way, “Somatic Symptoms”. The percentage of originally grouped cases that proved to have been correctly classified was 74.1%. The other dimensions did not improve the percentage of cases discriminated (Table 2).

Confirmation of the independence of the SCL-90-defined psychopathological profile from the level of the stress-related emotional condition came from a later study involving 93 heroin-addicted patients, divided into two samples according to the severity of their stress reaction and then allocated to one specific dimension based on their SCL-90 profile. While patients with a high response to stress, when they were compared with those who had a low level of response, showed higher psychopathological severity, no significant differences versus patients with a low response to stress were observed in the frequency of the five dimensions [22]. Lastly, we planned to ascertain whether the five-factor psychopathological configuration detected in heroin addicted patients could also connote cocaine- or alcohol-dependent patients too [23]. Looking at the overall sample of 2314 patients (449 alcohol-, 1195 heroin-, and 670 cocaine-dependent patients) entering a TC, patients with alcohol or heroin dependence, when compared with patients manifesting cocaine dependence, showed statistically significantly higher scores in the “Somatic Symptoms” dimension; patients with alcohol dependence exhibited statistically significantly higher scores than those with cocaine or heroin dependence in the “Panic Anxiety” dimension. Furthermore, a multinomial logistic regression showed a statistically significant negative association between the “Somatic-Symptoms” dimension and the cocaine versus the heroin group (OR 0.68; CL 95% 0.48–0.95) as well as a statistically significant positive association between the Sensitivity/Psychoticism dimension and the alcohol in comparison with the heroin group (OR 1.54; CL 95% 1.02–2.32). A further logistic regression, comparing cocaine and alcohol dependents, failed to identify any specific significant association with psychopathological dimensions. Even when polysubstance users were excluded, no differences in the frequency of the five psychopathological dimensions among heroin-, alcohol-, and cocaine-dependent patients were detected in a study involving 256 subjects with heroin, cocaine, or alcohol dependence [24]. It seems, therefore, that this five-factor structure characterizes both opioid dependents entering OAT and residential TC patients. It is not substantially modified by patients’ detoxification status, nor is it substantially modified by the presence of psychiatric problems. It distinguishes, with only minimal differences, alcohol and cocaine dependents, too. Thus, it seems to constitute a psychological/psychiatric trait rather than a state.

What now remains to be done is to verify whether this SCL-90-defined psychopathological structure is stable over time. In this connection, we carried out one study involving 636 patients who were evaluated at the baseline and after three months of TC residential treatment to estimate the magnitude of changes in severity and typology of their psychopathology [25]. The stability of the psychopathological typologies was evaluated by baseline–endpoint Cohen’s kappa. The results show a general reduction of SCL-90 severity, accompanied by a reduction in the frequency of the Somatic Symptoms and Worthlessness/Being Trapped dimensions, while the frequency of the Panic Anxiety and Violence/Suicide dimensions increased. A further report has been published on seven patients who stayed in OAT treatment for nearly 30 years. This report documented the presence of all the SCL-90 dimensions except for Worthlessness/Being Trapped [26].

To complement the results on the relationship between the SCL-90 psychopathological profile of people with heroin dependence and core components of addictive disorders, further research was conducted to verify the level of correlation of the five dimensions with the behavioral manifestations of addiction. This relationship was investigated, at the multivariate level, applying the canonical correlation analysis in a sample of 207 heroin addicts. In this group of patients, the severity of the “Violence/Suicide”, “Somatic Symptoms”, and “Panic Anxiety” dimensions on the one hand and the severity of craving-related areas of behavior (value assigned to a drug, control of use, risk exposure, and cue reactivity) on the other hand proved to be strongly associated (*p* < 0.001) [27].

A confirmation of the specificity of this five-dimensional structure of psychopathology for opioid addiction comes from comparisons carried out on other psychiatrically pertinent conditions. In this connection, we compared the SCL-90 five-factor psychopathological structure displayed by 972 heroin dependents with that shown by 504 patients with major depression. Three psychopathological dimensions (Somatic Symptoms, Worthlessness/Being Trapped, and Sensitivity/Psychoticism) turned out to be more frequent in heroin addicts, whereas Violence/Suicide proved to be better represented in depressed patients. When logistic regression was analyzed, including gender, age, and psychiatric severity, the SCL-90 psychopathological structure proved to be the best predictor of belonging to the heroin-dependent group of patients [28]. A comparison was also carried out versus patients with a psychotic disorder. In this case, 40 chronic psychotic patients were matched with 33 patients addicted to heroin, according to age and gender. In psychotic patients, Panic Anxiety was over-represented, whereas the Worthlessness/Being Trapped and Somatic Symptoms dimensions were under-represented. Multivariate discriminant analysis was able to significantly differentiate heroin addicts from psychotic patients [29].

To further support the psychopathology of addiction, we decided to verify whether the five SCL-90-based factors could discriminate heroin use-dependent (HUD) patients from other patients affected by a non-substance-related compulsive behavior, such as gambling (GD) and problematic Internet use (PIU). In the related study, we enrolled 972 heroin-dependent and 110 gambling patients. We then compared the two groups of patients, at the univariate and multivariate levels, for the frequency and the severity score of the five psychopathological dimensions. We observed that the Somatic Symptoms- and Violence/Suicide-related symptomatologies were more frequent in heroin-addicted patients, while Panic Anxiety-related symptoms were more frequent in gamblers. At the multivariate level, Somatic Symptoms turned out to be the dimension that was most discriminant, since it was sufficient to discriminate HUD from GD patients [30]. Regarding problematic Internet use (PIU), we applied the SCL-90 questionnaire to 493 youngsters, after subdividing them into two groups on the basis of the presence or absence of problematic Internet use (82 and 411 subjects, respectively). At the univariate level, all psychopathological dimensions were significantly higher in young people with PIU. At the multivariate level, the co-presence of high severity Somatic Symptoms and Panic Anxiety symptoms together discriminated—achieving a success rate of 98.2%—young people with PIU from PIU-free peers [31].

We then tested whether the psychopathology of substance-addicted patients, as ascertained by the five SCL-90 dimensions, could be differentiated from that of obese people. In this case, we explored the severity and frequency of the five SCL-90 dimensions, by comparing 972 patients with HUD and 106 obese individuals. Univariate and multivariate analyses revealed that the Worthlessness/Being Trapped, Somatic Symptoms, and Sensitivity/Psychoticism dimensions were more frequent among HUD patients, while the Panic Anxiety or Violence/Suicide dimensions were better represented in obese patients. SCL-90 dimensions were able to discriminate between the two groups. It is also worth noting that it was possible to reclassify 47.2% of obese subjects as HUD patients. This suggests the existence of an area of psychopathological overlap between the two conditions [32].

### 2.2. Potential Clinical Applications

**Figure 3:**
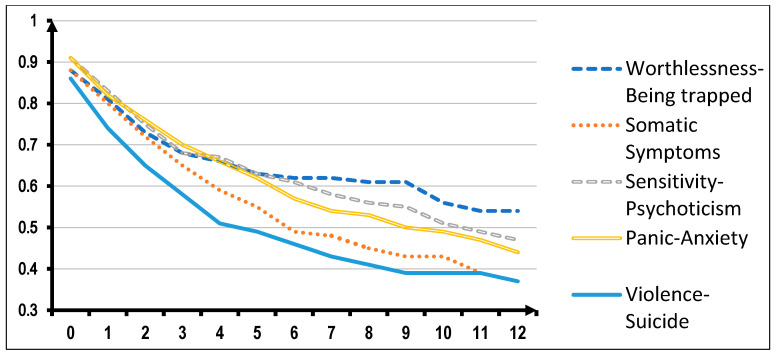
Survival in treatment of 2122 TC-treated patients according to the prominent psychopathology and independently of principal substance of abuse (Wilcoxon statistics = 17.69, DF = 4; *p* = 0.001).

Looking now at possible applications in clinical practice of this SCL-90-based structure, we verified its potential value in predicting the outcome of treatment. In this connection, we tested whether the five SCL-90 based psychopathological dimensions have any impact on the retention of patients in treatment. In that study, we included data obtained from answers to SCL-90 for the full sample of 2122 patients entering a TC. Retention in treatment was analyzed by applying the survival analysis and Wilcoxon statistics to compare the survival curves. What we observed was that patients with prevailing symptoms in the Worthlessness/Being Trapped dimension tend to stay significantly longer in treatment than those showing dominant symptoms in the other psychopathological dimensions. More specifically, prevailing symptoms in the “Violence/Suicide” dimension proved to be associated with a higher dropout rate (52.7% of entrants remaining in treatment after three months than the rate recorded in the Worthlessness/Being Trapped dimension (65.4% of entrants remaining in treatment after three months) (Figure 3).

In another study, we looked at the impact of the treatment with buprenorphine or methadone on the psychopathological profile of 213 patients with heroin addiction (106 treated with buprenorphine and 107 with methadone) in a follow-up study lasting 12 months [33,34]. Looking at the data for retention in treatment, patients belonging to the Sensitivity/Psychoticism group treated with methadone showed a significantly higher retention in treatment than those treated with buprenorphine; conversely, patients belonging to the Violence/Suicide group treated with buprenorphine showed a significantly higher retention in treatment than those treated with methadone.

## 3. Discussion

In this paper, we look at the relationship between addiction and concomitant psychopathology, reporting research evidence that supports an integrated view of behavioral manifestations of addiction and associated psychiatric symptoms. This unitary approach is coherent with the perspective adopted by the US National Institute of Mental Health with the RDoC system, which calls into question the categorical fragmentation of psychopathology inherent in the current nosographic systems (DSM and ICD), and, in contrast, encourages a dimensional research approach which, regardless of supposed boundaries between disorders, reconnects clinical manifestations with neurobiological processes. Consistent with this approach are lines of research oriented to construct a quantitative psychopathology based on the spontaneous aggregation of symptoms. In this sense the work carried out by the Hierarchical Taxonomy of Psychopathology (HiTOP; http://medicine.stonybrookmedicine.edu/HITOP) consortium deserves consideration [35]. The HiTOP consortium’s methodology includes the application of statistical methods, such as cluster analysis and exploratory/confirmatory factor analysis, to evaluate the degree of association of traits, signs, and symptoms shown by patients or even by the normal population. Of course, a quantitative nosology, oriented towards the recognition of statistically defined aggregations of symptoms that distinguish clinical manifestations, does not consider the neurobiological background; even so, it may be extremely useful in identifying clinically pertinent dimensions, which means phenotypes. This component can make a major contribution to RDoC research by connecting it up with etiology and pathophysiology.

At this point, we can attempt to clarify, in terms of neurobiology and pathophysiology, how symptoms belonging to these five dimensions may consistently be considered an integral component of the clinical presentation of addictive disorders.

### 3.1. Somatic Symptoms Dimension

According to our results, Somatic Symptoms were significantly more represented in patients entering opioid agonist treatment than in those entering a therapeutic community [16]. Moreover, Somatic Symptoms were significantly more represented in older opioid addicts [17], in those still involved in the active use of substances [20] and in opioid patients with lifetime psychiatric problems [21]. They were also significantly more represented in patients dependent on opioids than in those dependent on cocaine [23]. Lastly, somatic symptoms tend to decrease in patients who remains for three months in a TC [25] (Table 3).

The distinctive characteristics of this dimension may be easily understood by looking at SCL-90 items it includes. It may be noted that items comprised in “Somatic Symptoms” match typical complaints of the opioid withdrawal syndrome [36] (muscle aches, back pain, hot flushes, nausea, insomnia, etc.) [14]. As a result, the low scores recorded by DTX-patients belonging to this dimension can be explained by their low level of tolerance to opioids. Its association with withdrawal may also explain its lower pertinence in cocaine addicts, because of their usual low proneness to the physical components of withdrawal. The higher age of patients belonging to this dimension may depend on their longer history of addiction and the heavy burden of its somatic consequences. Even the discriminating contribution made by this dimension to the distinction of patients with lifetime psychiatric problems may be related to the high level of prevalence of somatic conditions accompanying psychiatric population [37,38,39,40,41,42,43] and the population of addicts who have dual diagnosis [44,45,46]. As this dimension is the one most strongly influenced by interfering variables, it can be considered a transient feature susceptible to modification through specific interventions, rather than a stable state reflecting the long-term psychopathological traits of persons affected with substance use disorder. The fall in the prevalence of this dimension after three months of abstinence from substances in TC is coherent with this transient nature.

### 3.2. Worthlessness/Being Trapped Dimension

This dimension does not seem to be influenced by the continued use of heroin versus detoxification treatment, or by the lifetime presence of psychiatric problems—not even by the kind of substance abused. It is, however, influenced by age (being more marked in older opioid addicts), by the setting of the treatment, and by the flow of time (Table 3). Its presence in opioid addiction can be better understood by considering the strong association between mood and substance use disorders, in terms of psychopathological risk factors, neurobiology, and epidemiology [47,48,49,50,51,52,53,54,55,56,57,58,59,60,61,62]. The presence, in individuals with substance use disorders, of symptoms such as worthlessness, sadness, and hopelessness about the future may be explained by biochemical changes observed in the reward, motivational, and inhibitory systems. We know that an amotivational outlook in patients with substance use disorder may be supported by long-term modifications in the reward and motivational system, including a steep fall in the dopaminergic tone, and the activation of the transcription factor CREB, with the consequently intensified expression of dynorphine [48,63,64,65,66,67,68,69,70,71]. The neuroendocrine stress system is likewise involved in the regulation of mood and in addictive behavior [72,73,74,75,76]. Our failure to find any association of this dimension with depression or bipolar spectrum disorders [17], together with the lack of any significant correlation with some aspects of craving-related behavior [27], is consistent with the presence of an inhibitory component in the reward deficiency syndrome [48]. The positive association of this dimension with access to OAT rather than to TC treatment calls into question the severity of addiction-related disruption to various areas of life (e.g., the family, work, and legal matters), with inevitable consequences on psychological well-being and the urgent need for recuperation, most easily obtained by OAT. Moreover, consistently with a manageable condition, this dimension seems to be influenced by the flow of time after detoxification, which, again, may link this dimension with the condition of active substance use [17].

### 3.3. Sensitivity/Psychoticism Dimension

The presence of symptoms belonging to this dimension in the clinical picture of patients with addictive disorders seems to be independent of sociodemographic factors, active opioid use, the presence of psychiatric problems, and even of craving-related behavior. On the other hand, it does seem to be affected by the kind of substance abused (alcohol) and by the time spent in a therapeutic community (Table 3). Symptoms of this dimension in opioid addicts, which distinguish about 20% of these patients, may result from the antipsychotic action of opioids [77,78,79,80,81,82,83]. It may also depend on a co-abuse of stimulants, cannabis, or alcohol, given their possible propsychotic action [84,85,86,87,88,89,90,91]. According to our results, Sensitivity/Psychoticism has been positively associated with dependence on alcohol, which may be accounted for by the psychotic feature of withdrawal from alcohol [92,93,94,95,96]. An alternative possible explanation for the Sensitivity/Psychoticism dimension might center on the unspecific rise in dopamine tone within the motivational system. This derangement has been put forward to explain psychotic conditions as the result of an indiscriminate attribution of salience [97,98]. The involvement of dynorphin and the k-opioid receptors, considering the well-known psychotomimetic effects of k-opioid agonists, should also be kept under review [63,99].

### 3.4. Panic Anxiety Dimension

This dimension is not associated with sociodemographic factors, active heroin use or detoxification status, the setting of the treatment (OAT versus TC), the kind of substance abused, or the time spent in the therapeutic community. Moreover, it is positively associated with the presence of lifetime psychiatric problems and with craving-related behaviors. This dimension therefore seems to be a quite stable component of the opioid addiction picture, and to be independent of most of the confounding factors, while remaining closely linked to the core behavioral components of opioid addiction (Table 3). Its presence in addicted people may be explained by the persistence in chronic opioid dependents of what is often called “protracted withdrawal”, with symptoms such as malaise, irritability, dysphoric mood, emotional pain, and stress reactivity, which constitute an essential component of the negative emotional state that distinguishes the long-term psychopathological condition of substance use dependents. This condition seems to be related to the elevation of reward thresholds, resulting from a fall in dopamine tone, which persists even after the interruption of substance use [100,101,102,103], as well as related to the activation of the neuroendocrine stress system, which is involved in modulation of affectivity and addiction-related behavior [72,73,74,75,76] Given the transnosographic character of anxiety-related signs and symptoms, it should not be surprising that it is most frequently displayed by people with lifetime psychiatric problems, even though it should also be stressed that this dimension does not prove to be capable of identifying cases of major depression.

Interestingly, the Panic Anxiety dimension does not seem to be influenced by the length of a patient’s stay in TC treatment, considering that even after three months its related symptoms do not subside. The general tendency for anxiety symptoms to persist might depend on their existence before the onset of substance use, as the result of inherited or acquired vulnerability, probably involving the neurocircuits already named above; otherwise, it might have been the product of persistent neural changes triggered by addictive processes.

### 3.5. Violence/Suicide Dimension

This dimension is significantly associated with being an outpatient receiving OAT treatment and with dropping out from a TC residential program. It does not, however, turn out to be associated with other factors explored in our studies, including time spent in a therapeutic community (Table 3). This dimension is distinguished by impulsivity. The existence of strong interconnections between impulsive and addictive behavior is supported by the involvement of the same brain regions and neurocircuits of the motivational and inhibiting-control systems, as well as the same precursors and vulnerabilities, such as novelty seeking, reckless behaviors, disinhibition, and antisociality [104,105,106,107,108,109,110,111,112,113,114,115,116,117,118,119,120]. The association with OAT outpatients could be due to the fact that symptoms included in this dimension may find more appropriate responses in the medical expertise made available in an OAT. This dimension also proves to be closely connected with the core behavioral alterations that are related to craving.

Summarizing the results of our research on the SCL-90-based psychopathology of opioid addiction, it seems that a dimension such as Somatic Symptoms and, within a narrower range, Worthlessness/Being Trapped, which are those displayed by about 30–35% of patients involved in our studies, are more closely associated with the active use of substances and a consequent lifestyle. Therefore, symptoms belonging to these two dimensions may be more dependent on the acute psychic effects of substances and on more reversible changes in the neurobiological addictive processes, as well as on the chances of ending the disruptive style of life, which almost always accompanies dependence on substances of abuse. Two other dimensions, Panic Anxiety and Violence/Suicide, representing about 42% of patients, are strictly tied to craving-related behavior; do not seem to be substantially influenced by interfering factors; and tend to persist irrespectively of abstinence from the use of substances. Their persistence may depend on the low-likelihood reversibility or the absolute irreversibility of changes in the neural systems involved in addictive processes, as well as being conditioned by the persistence of psychological/psychiatric vulnerabilities that precede the use of substances. These dimensions may be more closely associated with the “core” pathophysiological processes of addiction, being part of the psychopathological features that predispose to addiction, as well as to the more persistent psychopathological changes that ensue from addiction. The same observation is not appropriate to the Sensitivity/Psychoticism dimension, which marks out the remaining 20% of patients. Although it is not influenced by most of the confounding factors considered, it does not turn out to be associated with craving-related behaviors.

### 3.6. Limitations

Our results on the psychopathological structure of opioid addiction were obtained exclusively through detailed analysis of the SCL-90 questionnaire. SCL-90 offers the advantage of easy administration and the short time required for its completion. The only problem created by this procedure is that it essentially relies on the subjective judgment of patients, which may negatively affect the quality of their insights. The aim of ensuring a correct understanding of the questions, thus avoiding the tendency to manipulate the answers and reinforcing the motivation for participating into the study, would have required the use of further instruments.

We also have to consider the limited range of behavior, traits, and symptoms explored by SCL-90. We are aware that the use of more appropriate instruments would have made it possible to investigate domains closely connected with the neurobiological processes involved in addiction (e.g., reward, impulse control, and stress systems). We have actually chosen this second path in investigating the area of stress-related symptoms, using more appropriate instruments and observing a more specific clinical expression in opioid addicts [22,121].

Besides limitations to the generalizability of results due to the exclusive use of SCL-90 as measure of psychopathology, we have to consider that most of our studies on the psychopathology of addiction have been carried out on large clinical samples (not selected by screening criteria) of Italian patients dependent on opioids and about to enter outpatient OAT or TC. Although the consistency of the results obtained with these two populations and their congruity in comparison with other populations (chosen according to substance(s) used, patients’ nationality, and a further diagnosis) have been verified, confirmations coming from other countries, other settings, and other substances of abuse would certainty increase their validity.

## 4. Conclusions

### 4.1. From the Research Viewpoint

Research is going forward in trying to understand the connection between clinical manifestations of addiction and other mental disorders by exploring the involved neurobiological structures and processes, and in this way trying to construct a new nosography based on pathophysiological and etiopathogenic roots. In this connection, the identification of psychopathological dimensions resulting from the spontaneous association of psychological/psychiatric features manifested by patients with addictive disorders should help in the task of defining pertinent links with underlying shared neurobiological processes. Research on the psychopathology of addiction should consider applying methods such as cluster analysis and exploratory/confirmatory factor analysis using SCL-90 and other tests/questionnaires that may be useful in bringing consistency to a specific definition of psychopathological dimensions that distinguish opioid consumption and other forms of dependence. The evidence made public by our studies would need confirmation from studies carried out in other countries, settings, and populations, which look at a variety of different sociodemographic conditions, substances of abuse, and treatments. Moreover, a longitudinal approach would require the application of methods based on cluster analysis and exploratory factor analysis to the psychological/psychiatric features of subjects considered to be passing through a particular stage of the progression of their illness: vulnerability phase; active use phase; and full-blown addiction. Finally, the predictive value of the SCL-90 classification of psychopathology calls for deep exploration, in relation to specific treatments and outcomes (retention in treatment, use of substances, craving, and psychological/psychiatric well-being).

### 4.2. From the Clinical Viewpoint

From a clinical perspective, we have to consider the need to evaluate the extended range of the psychological symptoms that accompany addictive clinical presentations. These may be strong or mild symptoms that do not necessarily deserve to be treated as comorbidity. Often, they start early in preadolescence as a risk factor for the encounter with substances of abuse and subsequent escalation to addiction. A longitudinal approach to their evaluation will yield a unitary view of the psychiatric implications for the treatment, primarily based on the instruments of addictionology; only if those symptoms fail to subside should that case be considered as an example of psychiatric comorbidity. The use of SCL classifications may be useful in following the clinical routine, by allowing a better understanding of clinical presentations, supporting clinicians in making prognostic evaluation, and getting the right match between patients and treatments. Care providers should consider the advantages of acquiring psychiatric expertise that is likely to prove useful in implementing the SCL-90 classification in the treatment program, in terms of both care quality improvement and program outcome.

## Figures and Tables

**Table 1:** Differences in severity of psychopathological dimensions between non-detoxified (NDTX) and detoxified (DTX) heroin-dependent subjects: stepwise discriminant analyses.

Steps	Psychopathological Dimensions	NDTX Patients	DTX Patients	F	*p*	DF *
		*N* = 641	*N* = 347			
1	Somatic Symptoms	1.12 ± 0.7	0.77 ± 0.7	34.14	0.000	1.34
	Worthlessness/Being Trapped	1.25 ± 0.8	0.95 ± 0.7	53.25	0.000	
	Sensitivity/Psychoticism	0.83 ± 0.7	0.68 ± 0.6	11.41	0.001	
	Panic Anxiety	0.48 ± 0.6	0.33 ± 0.5	13.91	0.000	
	Violence/Suicide	0.89 ± 0.7	0.61 ± 0.6	37.30	0.000	
	Centroids	0.17	−0.22			

* Discriminant Function; Multivariate statistics: Wilks’ Lambda = 0.95; Chi-squared = 51.87, DF = 1; *p* = 0.000; 64.4% of originally grouped cases were correctly classified.

**Table 2:** Differences in severity of psychopathological symptoms between opioid dependent subjects with (PC) and without (NPC) ascertained lifetime psychiatric problems at TC treatment onset: stepwise discriminant analyses.

Steps	Psychopathological Dimensions	NPC-HA Patients	PC-HA Patients	F	*p*	DF *
		*N* = 886	*N* = 309			
1	Panic Anxiety	0.35 ± 0.5	0.64 ± 0.7	51.65	<0.001	0.71
2	Somatic Symptoms	0.92 ± 0.7	1.25 ± 0.8	43.28	<0.001	0.37
	Worthlessness/Being Trapped	1.05 ± 0.7	1.37 ± 0.8	36.78	<0.001	
	Sensitivity-Psychoticism	0.70 ± 0.6	0.97 ± 0.7	38.13	<0.001	
	Violence-Suicide	0.71 ± 0.6	0.99 ± 0.8	37.21	0.001	
	Centroids	−0.12	0.37			

* Discriminant function: Wilks’ Lambda = 0.95; Chi-squared = 55.83, DF = 2; *p* < 0.001; 74.1% of originally grouped cases had been correctly classified.

**Table 3:** Statistically significant associations between SCL-90-based psychopathological dimensions that are specific to substance use disorder and other demographic and clinically pertinent variables.

	Statistically Significant Associations							
SCL-90 Dimensions	Age	Setting AOT vs. TC	Active Use	Craving	Lifetime Psychiatric Problems	Kind of Substance		Instability Over Time
						Alcohol	Cocaine	
Worthlessness/Being Trapped	•	•						
Somatic Symptoms	•		•	•	•		•	•
Sensitivity/Psychoticism						•		
Panic Anxiety				•	•			
Violence/Suicide		•		•				
